# The Mitochondrial T16189C Polymorphism Is Associated with Coronary Artery Disease in Middle European Populations

**DOI:** 10.1371/journal.pone.0016455

**Published:** 2011-01-26

**Authors:** Edith E. Mueller, Waltraud Eder, Sabine Ebner, Eva Schwaiger, Danijela Santic, Tanja Kreindl, Olaf Stanger, Bernhard Paulweber, Bernhard Iglseder, Hannes Oberkofler, Richard Maier, Johannes A. Mayr, Franz Krempler, Raimund Weitgasser, Wolfgang Patsch, Wolfgang Sperl, Barbara Kofler

**Affiliations:** 1 Department of Pediatrics, Paracelsus Medical University, Salzburg, Austria; 2 Department of Cardiac Surgery, Paracelsus Medical University, Salzburg, Austria; 3 Department of Internal Medicine, Paracelsus Medical University, Salzburg, Austria; 4 Department of Geriatrics, Paracelsus Medical University, Salzburg, Austria; 5 Department of Laboratory Medicine, Paracelsus Medical University, Salzburg, Austria; 6 Department of Ophthalmology, Medical University Graz, Graz, Austria; 7 Department of Internal Medicine, Hospital Hallein, Hallein, Austria; University of Tor Vergata, Italy

## Abstract

**Background:**

The pivotal role of mitochondria in energy production and free radical generation suggests that the mitochondrial genome could have an important influence on the expression of multifactorial age related diseases. Substitution of T to C at nucleotide position 16189 in the hypervariable D-loop of the control region (CR) of mitochondrial DNA (mtDNA) has attracted research interest because of its suspected association with various multifactorial diseases. The aim of the present study was to compare the frequency of this polymorphism in the CR of mtDNA in patients with coronary artery disease (CAD, n = 482) and type 2 diabetes mellitus (T2DM, n = 505) from two study centers, with healthy individuals (n = 1481) of Middle European descent in Austria.

**Methodology and Principal Findings:**

CR polymorphisms and the nine major European haplogroups were identified by DNA sequencing and primer extension analysis, respectively. Frequencies and Odds Ratios for the association between cases and controls were calculated. Compared to healthy controls, the prevalence of T16189C was significantly higher in patients with CAD (11.8% vs 21.6%), as well as in patients with T2DM (11.8% vs 19.4%). The association of CAD, but not the one of T2DM, with T16189C remained highly significant after correction for age, sex and body mass index (BMI) and was independent of the two study centers.

**Conclusions and Significance:**

Our results show for the first time a significant association of T16189C with CAD in a Middle European population. As reported in other studies, in patients with T2DM an association with T16189C in individuals of European decent remains questionable.

## Introduction

Mitochondria produce energy to sustain cell growth and function, but they also generate harmful free radicals and mediate apoptosis. Mutations of mitochondrial DNA (mtDNA) can cause diseases such as MELAS (mitochondrial encephalomyopathy, lactic acidosis and stroke-like episodes) and Leber's hereditary optic neuropathy. In rare cases of type 2 diabetes mellitus (T2DM), pathogenic mtDNA mutations have been linked to the disease. The most frequent mtDNA mutation found to be associated with enhanced risk of diabetes is A3234G, which affects the tRNA^Leu^ gene. When present in a high percentage (>85%) of genomes, the mutation often leads to MELAS. When A3234G is present at lower heteroplasmy (5% to 30%), the mutation is associated with maternally inherited diabetes mellitus and deafness (MIDD), accounting for 0.2–2% of diabetes cases [1], [2].

In addition, there is evidence that “neutral” polymorphisms in mtDNA may actually be risk factors for age related multifactorial diseases and can influence disease outcome. The T to C substitution at position 16189 within the regulatory displacement loop (D-loop) of mtDNA is such a polymorphism, with reports of associations with a variety of multifactorial disorders [3]–[13]. This substitution (referred to in the literature as the “16189 variant”) frequently produces an uninterrupted polycytosine (poly-C) tract in the D-loop. Because polynucleotide tracts are susceptible to replication errors, heteroplasmic (more than one distinct population of mtDNAs in the same individual) length variation of such uninterrupted poly-C tracts is frequently observed [11], [14].

Associations of the 16189 variant have been found for metabolic syndrome [9], dilated cardiomyopathy [11], left ventricular hypertrophy [12], lacunar cerebral infarction [13], hereditary haemochromatosis [15], reduced birth weight, lower ponderal index, and high placental-to-birth weight ratio as well as thinness in young adults [16]–[18]. The 16189 variant was also reported to be associated with higher fasting insulin and glucose levels, insulin resistance, and/or T2DM in Europe [3], [19], [20] and Asia [4]–[9], [21]. However, other studies failed to find significant associations between the 16189 variant and T2DM or metabolic phenotypes in the United Kingdom [14], [22] and Finland [23].

Recently, an association of T16189C to coronary artery disease (CAD) was reported in Arabs of Saudi descent [10].

Mitochondria are likely to be important for insulin secretion in pancreatic β cells. In the ATP-sensitive K^+^ channel (K_ATP_)-dependent pathway of insulin secretion, oxidative phosphorylation in mitochondria leads to ATP production and consequently to a rise in the ATP:ADP ratio. This event directs closure of the K_ATP_, resulting in depolarisation of the plasma membrane, opening of voltage-gated Ca^2+^ channels, and finally to exocytosis and insulin secretion [24]. Mitochondrial generation of ATP may be linked to glucose sensing through interaction with β cell glucokinase. Therefore, mtDNA mutations (or reduced mtDNA content) that result in lower levels of ATP production may influence both insulin secretion and glucose sensing, thus contributing to insulin resistance [25]. Lower mtDNA copy number in peripheral blood mononuclear cells was shown to precede the development of diabetes [26].

It has been demonstrated that enhanced production of reactive oxygen species (ROS), which are generated predominantly in mitochondria, is a common feature of risk factors for atherosclerosis. ROS are central to some of the most important processes implicated in atherogenesis, including dysfunction and apoptosis of endothelial cells, activation of matrix metalloproteinases, growth of vascular smooth muscle cells and their migration into the intimal layer, expression of adhesion molecules, and oxidation of low density lipoproteins [27], [28]. Hence, mtDNA mutations that alter the production of ROS might contribute to the generation of CAD.

The aim of the present study was to compare the frequency of T16189C as well as other polymorphisms in the control region (CR) of mtDNA from patients with T2DM and CAD versus healthy controls in Middle Europe.

## Results

Clinical characteristics of the patients and controls are shown in Table 1.

**Table 1:** Characteristics of the study populations.

	Controls	CAD^a^		T2DM^b^	
		Graz	Salzburg	Graz	Salzburg
	n = 1481	n = 191	n = 291	n = 226	n = 279
Mean (SD^c^) age (years)	51.5 (6.1)	53.7 (5.5)	69.8 (9.4)	73.1 (11.0)	62.3 (11.6)
Male (%)	64.3	90.6	68.0	40.7	57.0
Mean (SD^c^) BMI^d^ (kg/m^2^)	26.7 (4.1)	27.0 (3.2)(189)	27.4 (4.0)	27.6 (5.0)	30.1 (5.5)
History of myocardial infarction (%)	0	62.8	41.2	12.4	n.a.^e^
Diagnosis of diabetes (%)	0	12.0	34.7	100	100
Hypertension (%)	13.8(1372)	49.2	79.0	73.0	54.9

a CAD  =  coronary artery disease.

b T2DM  =  type 2 diabetes mellitus.

c SD  =  standard deviation.

d BMI  =  body mass index.

e n.a.  =  not available.

Missing values represent less than 10% of the total, with the exception of BMI data in patients with T2DM from Graz and hypertension data in patients with T2DM from Salzburg.

Because there are several studies reporting contradictory findings as to the association of the 16189 variant and T2DM, we first analyzed the frequency of T16189C in our patient and control groups. We found an elevated frequency of T16189C in patients with T2DM compared to healthy controls (Table 2). In patients with CAD we also found an elevated frequency of T16189C compared to healthy controls (Table 2).

**Table 2:** Frequencies (%) of the CR polymorphism T16189C and the poly-C tract in cases and controls as well as the corresponding Odds Ratios and 95% Confidence Intervals.

	Controls	CAD^a^	P-Value^c^(Pearson chi-square test)	OR^d^(95% CI^e^)	aOR^f^(95% CI^e^)	T2DM^b^	P-Value^c^(Pearson chi-square test)	OR^d^(95% CI^e^)	aOR^f^(95% CI^e^)
	n = 1481	n = 482				n = 505			
T16189C	11.8	21.6	<0.0000001	2.05 (1.6–2.7)	2.17 (1.5–3.1)***	19.4	<0.00005	1.80 (1.4–2.4)	1.46 (1.0–2.2)
Poly-C tract	9.1	15.8	<0.00005	1.87 (1.4–2.5)	1.61 (1.1–2.5)*	15.4	<0.0001	1.82 (1.4–2.5)	1.45 (0.9–2.3)

a CAD  =  coronary artery disease.

b T2DM  =  type 2 diabetes mellitus.

c P-value: compared to controls.

d OR  =  Odds Ratio.

e CI  =  Confidence Interval.

f adjusted for age, sex and body mass index.

*p<0.05, p>0.01.

***p<0.001.

**Figure 1:**
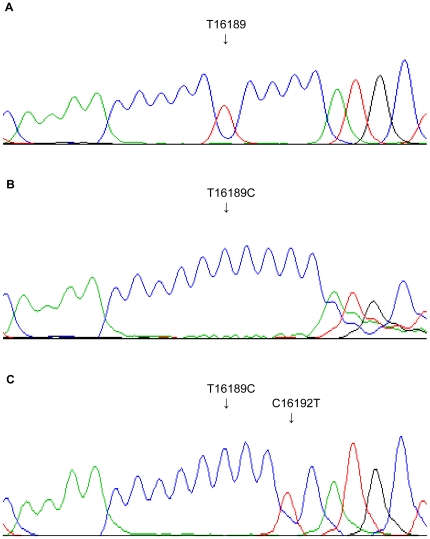
MtDNA CR sequence variations around position 16189. **A**) Wild-type sequence. **B**) T16189C polymorphism, causing a poly-C tract with heteroplasmic length variation. **C**) T16189C polymorphism with an additional polymorphism at position 16192, interrupting the poly-C tract.

We next analyzed the frequency of the poly-C tract. A poly-C tract is generated when the wild type tyrosine in position 16189 of the mtDNA is substituted by a cytosine (T16189C) resulting in a polynucleotide tract of at least 10 cytosines (Figure 1B). Frequently, the poly-C tract also leads to a heteroplasmic length variation, causing varying lengths of the poly-C tract (>10 cytosines) in different mtDNA molecules of one subject (Figure 1B). However, the T16189C polymorphism can be accompanied by a second mutation for example at position 16192, interrupting the poly-C tract (Figure 1C). In this manuscript, the term poly-C tract was used for CR sequences with the T16189C polymorphism and an uninterrupted poly-C tract of at least 10 cytosine residues. For instance, the CR sequence illustrated in Figure 1C was regarded as T16189C, but not as a poly-C tract, whereas the sequence illustrated in Figure 1B was taken into account in both groups. The frequency of the poly-C tract was significantly higher in patients with CAD and T2DM compared to the control population (Table 2).

When patients with diabetes were excluded from the CAD cohort (n = 358), there was an even greater difference in the frequency of T16189C and the poly-C tract between the CAD and the control group [T16189C: 22.1% vs. 11.8%, p<0.000001, OR 2.11 (1.6–2.8); poly-C tract: 16.2% vs. 9.1%, p<0.0001, OR 1.93 (1.4–2.7)].

Using multiple regression analysis, the association between T16189C and the poly-C tract and CAD remained significant after correction for age, sex and BMI (T16189C: p<0.00005; poly-C tract: p<0.05; Table 2). Because of the substantial age difference of patients with CAD and controls, we stratified the control group by age. For this purpose we selected the oldest individuals from the control group (age ≥57 years, n = 322). When the frequencies of T16189C and the poly-C tract in patients with CAD were compared to this control group (mean age 60 years) with a similar mean of age as the patients with CAD (mean age 63 years), the difference of the frequencies remained significant [T16189C: 21.6% vs. 12.7%, p<0.005, OR 1.89 (1.3–2.8); poly-C tract: 15.8% vs. 9.3%, p<0.01, OR 1.82 (1.2–2.9)].

Moreover, the association between T16189C and the poly-C tract and CAD was very similar in both study centers, Salzburg and Graz (Table 3).

**Table 3:** Frequencies (%) of the CR polymorphism T16189C and the poly-C tract in patients with coronary artery disease from Graz and Salzburg and controls as well as the corresponding Odds Ratios and 95% Confidence Intervals.

	Controls	CAD^a^ Graz	P-Value^b^(Pearson chi-square test)	OR^c^(95% CI^d^)	aOR^e^(95% CI^d^)	CAD^a^ Salzburg	P-Value^b^(Pearson chi-square test)	OR^c^(95% CI^d^)	aOR^e^(95% CI^d^)
	n = 1481	n = 191				n = 291			
T16189C	11.8	22.0	<0.0001	2.10 (1.4–3.1)	2.17(1.4–3.3)***	21.3	<0.00005	2.02 (1.5–2.8)	2.08 (1.1–3.9)*
Poly-C tract	9.1	14.7	<0.05	1.71 (1.1–2.7)	1.58 (1.0–2.5)	16.5	<0.0005	1.97 (1.4–2.8)	1.58 (0.8–3.2)

a CAD  =  coronary artery disease.

b P-value: compared to controls.

c OR  =  Odds Ratio.

d CI  =  Confidence Interval.

e adjusted for age, sex and body mass index.

*p<0.05, p>0.01.

***p<0.001.

Because a recent publication of our laboratory reported mitochondrial haplogroup T to be significantly overrepresented in CAD [29], we evaluated a possible association between haplogroup T and T16189C as well as the poly-C tract. There was linkage between haplogroup T and T16189C, but not between the haplogroup and the poly-C tract. However, this linkage was only partial, as the Pearson correlation coefficient was low (r^2^ (T16189C): 0.166). However, the association between T16189C and the poly-C tract and CAD was independent of mitochondrial haplogroup T. Evidence, that the frequency of T16189C was independent of haplogroup T is that T16189C and the poly-C tract were also significantly associated with CAD in non-haplogroup T individuals (Table 4).

**Table 4:** Frequencies (%) of the CR polymorphism T16189C and the poly-C tract in cases and controls with mitochondrial haplogroups other than T as well as the corresponding Odds Ratios and 95% Confidence Intervals.

Subjects with haplogroups other than T	Controls	CAD^a^	P-Value^c^(Pearson chi-square test)	OR^d^(95% CI^e^)	aOR^f^(95% CI^e^)	T2DM^b^	P-Value^c^(Pearson chi-square test)	OR^d^(95% CI^e^)	aOR^f^(95% CI^e^)
	n = 1355	n = 411				n = 455			
T16189C	10.5	19.0	<0.00001	2.00 (1.5–2.7)	1.95 (1.3–3.0)**	16.5	<0.001	1.69 (1.2–2.3)	1.38 (0.8–2.3)
Poly-C tract	8.7	17.0	<0.000005	2.15 (1.6–3.0)	2.03 (1.3–3.2)**	15.2	<0.0001	1.87 (1.4–2.6)	1.46 (0.9–2.4)

a CAD  =  coronary artery disease.

b T2DM  =  type 2 diabetes mellitus.

c P-value: compared to controls.

d OR  =  Odds Ratio.

e CI  =  Confidence Interval.

f adjusted for age, sex and body mass index.

**p<0.01, p>0.001.

In the case of T2DM however, both T16189C and the poly-C tract lost their significant association with the disease after correction for age, sex and BMI (T16189C: p = 0.08; poly-C tract: p = 0.13) (Table 2). After stratification of the disease populations according to the study center, the association of T16189C with T2DM remained significant after correction with age, sex and BMI when the control population was compared to patients from Graz (p<0.01) (Table 5), but not when the control population was compared to patients from Salzburg (p = 0.23) (Table 5).

**Table 5:** Frequencies (%) of the CR polymorphism T16189C and the poly-C tract in patients with type 2 diabetes from Graz and Salzburg and controls as well as the corresponding Odds Ratios and 95% Confidence Intervals.

	Controls	T2DM^a^Graz	P-Value^b^(Pearson chi-square test)	OR^c^(95% CI^d^)	aOR^e^(95% CI^d^)	T2DM^a^ Salzburg	P-Value^b^(Pearson chi-square test)	OR^c^(95% CI^d^)	aOR^e^(95% CI^d^)
	n = 1481	n = 226				n = 279			
T16189C	11.8	20.8	<0.0005	1.96 (1.4–2.8)	3.45 (1.4–8.3)**	18.3	<0.005	1.67 (1.2–2.4)	1.33 (0.8–2.1)
Poly-C tract	9.1	15.0	<0.01	1.77 (1.2–2.6)	2.05 (0.7–5.6)	15.8	<0.001	1.87 (1.3–2.7)	1.43 (0.9–2.4)

a T2DM  =  type 2 diabetes mellitus.

b P-value: compared to controls.

c OR  =  Odds Ratio.

d CI  =  Confidence Interval.

e adjusted for age, sex and body mass index.

**p<0.01, p>0.001.

We also compared the frequencies of other polymorphisms of the mtDNA CR (besides T16189C) between the control and disease populations. Polymorphisms with a p-value lower than 0.05 were examined for associations with T16189C and haplogroup T. When T2DM patients were compared to controls, A16183C and T16224C were found to be significantly associated with the disease; however, both also showed significant associations with T16189C. When CAD patients were compared to controls, T16183C, C16294T, T16304C, A73G and T195C were significantly associated with the disease. However, T16183C, C16294T, A73G and T195C also showed associations with T16189C, and T16304C is linked to mitochondrial haplogroup T (subhaplogroup T2) (Supplementary Table S1 and S2) [29].

The mitochondrial haplogroup frequencies in the controls, the CAD patients, and the T2DM patients are shown in Table 6. As reported previously, mitochondrial haplogroup T is more frequent in CAD whereas haplogroup H is underrepresented [29]. In the case of patients with T2DM, no significant differences were observed between their mtDNA haplogroup frequencies and those of the control population.

**Table 6:** Frequencies (%) of mitochondrial haplogroups in cases and controls.

Haplogroup	Controls^d^	CAD^ad^	T2DM^b^
	n = 1481	n = 482	n = 505
H	43.7	37.3	42.6
U	15.5	15.4	14.6
J	11.5	11.0	9.3
T	8.5	14.7	9.9
K	5.3	2.9	4.3
W	2.1	1.9	1.6
V	1.7	3.1	3.2
I	0.9	1.3	1.8
X	1.3	3.1	3.0
Others^c^	9.5	9.3	9.7

a CAD  =  coronary artery disease.

b T2DM  =  type 2 diabetes mellitus.

c Haplogroups that could not be assigned to one of the nine major European haplogroups by the SNP combination.

d Data on haplogroups have been reported recently [29].

## Discussion

In Asia, where the incidence of diabetes is growing rapidly under the influence of a Western lifestyle, several studies have reported that the 16189 variant is associated with multifactorial diseases such as diabetes (and its risk factors), metabolic syndrome, left ventricular hypertrophy and lacunar cerebral infarction [4]–[6], [8], [9], [12], [13], [21]. In Europe, however, the published data are conflicting [3], [11], [14]–[20], [22], [23]. A reason for this discrepancy might be that the prevalence of the T16189C polymorphism is higher in Asia than in Middle Europe, and that the power of a polymorphism to influence disease outcome is dependent on its frequency in the population [30].

Indeed, in the present study we were able to find an elevated frequency of T16189C and the poly-C tract in patients with T2DM compared to controls. This association has to be viewed with caution however, as the significant association was lost after correction for age, sex and BMI. When the two study centers Graz and Salzburg were analyzed independently, the association of T2DM and T16189C was found to be significant in Graz, but not in Salzburg, after applying adjustment for age, sex and BMI. However, as the control subjects were recruited at the University Hospital Salzburg, also the significant association of T16189C with T2DM in Graz is questionable. Age was the most prominent factor in reducing significance after correction. We don't know what other hidden confounders might have contributed to the changed outcome of the model, however.

In contrast, in the present study we were able to show a significant association of T16189C and the poly-C tract in the CR of the mitochondrial genome with CAD in a Middle European population for the first time. This association is independent of mitochondrial haplogroup T and stable, also after correction for age, sex and BMI and when analyzing the two study centers separately. The associations of the poly-C tract and CAD are less pronounced, but this seems to be only the result of a smaller sample size, as the Odds Ratios are stable as well.

Recently, Abu-Amero *et al* detected a significantly higher prevalence of T16189C (p = 0.017, OR 1.524 (1.1–2.2)) and the poly-C tract (p = 0.041) in CAD patients (T16189C: 27.8%) compared to controls (T16189C: 20.2%) in Saudi Arabs. In their study, 669 patients with angiographically documented CAD were compared to 258 control subjects. They reported that the association of T16189C with CAD was influenced by age and the presence of myocardial infarction [10]. Indeed, we also found age to be a primary determinant in the reduction or loss of significance in the association between this CR polymorphism and CAD or T2DM in multiple regression analysis (Table 2, 3, 4 and 5). To exclude an influence of age on the observed association, age stratification of controls was performed and revealed again a significant association of CAD with T16189C. Furthermore, an independent control group (n = 200) recruited at the Department of Ophthalmology (Paracelsus Medical University, Salzburg, Austria), with an average age of 75 years, shows nearly identical frequencies of T16189C and the poly-C tract as the control group used in this study (data not shown), indicating that age does not influence the frequency of these variants.

Limitations of the present study are that the case and control groups were not age- and sex-matched, and that the disease groups from Salzburg and Graz were compared to the same control group from Salzburg. However, our sample sizes were large (T2DM n = 505, CAD n = 482, controls n = 1481), the two study centers are less than 200 miles apart and only included individuals of Middle European descent. Therefore, we do not expect that there are significant differences in the frequency of mtDNA variations in healthy individuals between the two study centers.

In the literature, associations of the 16189 variant in the CR of mtDNA with diseases have been hypothesized to result from altered protein binding in this region, subsequently leading to changed mtDNA replication [3]. Recently, Park *et al* observed that the mitochondrial single-strand DNA-binding protein (mtSSB) bound less efficiently to mtDNA having the 16189 variant compared to mtDNA with the wild type sequence. This protein functions in stabilising the D-loop and in maintaining mtDNA. A novel origin of replication was found close to 16189 in the mitochondrial CR, suggesting a model in which the 16189 variant could reduce mitochondrial replication and thereby mtDNA content of the cell through altered binding affinity of mtSSB [6]. Moreover, heteroplasmic cybrid cell lines generated by fusion of mtDNA-free 143B cells with platelets from a patient who was heteroplasmic for the 16189 variant quickly lost the mutant mtDNA in culture. This indicates a reduced mtDNA replication rate in cells with the 16189 variant [31].

Reduced mtDNA content could also affect the efficiency of the electron transport chain, lower the ATP:ADP ratio, and enhance the formation of ROS. An increase in ROS would cause mitochondrial damage and further dysfunction of the electron transport chain, initiating a vicious circle [32]. These two features, lower ATP:ADP ratio and increased ROS production, could contribute to the onset of the multifactorial diseases diabetes and CAD.

Moreover, Lin *et al* suggested that, in a diabetic state, patients carrying the 16189 variant might not be able to cope in the same way with oxidative damage as patients without the variant [33]. A similar mechanism might be operative in patients with CAD.

Taken together, we propose a role for T16189C and the poly-C tract in the complex aetiology of CAD in Austria. The role of T16189C in T2DM remains unclear.

In CR polymorphisms other than T16189C and in haplogroups other than T, no novel significant differences were found between controls and patients with T2DM or CAD (a significantly higher haplogroup T in CAD was published elsewhere [29]). Two recent large studies also were not able to detect an influence of mitochondrial haplogroups and polymorphisms in the development of diabetes [34], [35]. On the other hand, T2DM was found to be associated with mitochondrial haplogroups J and T and with polymorphisms T4216C and A4917G (in linkage with J and T), in Caucasian-Brazilian patients [36].

As inter-study results for the same mitochondrial polymorphisms or haplogroups differ, it has to be kept in mind that the diseases analysed are multifactorial. Associations with nuclear polymorphisms and/or environmental factors not accounted for in the study design could bias the outcome of the analysis. Regional variation of polymorphism frequencies interacting with regional variation of lifestyle habits such as diet and exercise could make such contrasting findings possible.

Taken together, our findings reported here support previous observations that T16189C has detrimental effects on mitochondrial function in CAD and possibly also in T2DM.

## Methods

### Ethics Statement

The study was conducted according to the Austrian Gene Technology Act and complied with the Declaration of Helsinki. All subjects gave written informed consent before entering the study. The SAPHIR program was approved by the Local Province of Salzburg Ethics Committee (“Ethikkommission für das Bundesland Salzburg; Amt der Salzburger Landesregierung, Abteilung 9 Gesundheit und Sport”).

### Study subjects

Data from 2468 Caucasian subjects from central (University Hospital Salzburg) and southern (University Hospital Graz) Austria were analysed. Patients with CAD were recruited at the University Hospitals Salzburg (n = 291) and Graz (n = 191). All CAD patients had angiographically documented CAD with at least one of the main coronary arteries showing ≥50% stenosis. Patients with T2DM were recruited from the diabetes outpatient clinics of the University Hospital Salzburg and the Salzburg county Hospital Hallein (n = 279), and from the University Hospital Graz (n = 226).

The control population consisted of 1481 unrelated individuals, as previously described in detail [37]. Briefly, the cohort comprised 953 men between 39 and 66 years of age and 528 women between 39 and 67 years who were recruited to the Salzburg Atherosclerosis Prevention Program (SAPHIR) [37]. Exclusion criteria for participation in this study were a history of CAD, heart failure, cerebrovascular disease, peripheral vascular disease, haemodynamically relevant heart valve disease, chronic disease (of the liver or kidney, autoimmune disorders, malignant cancer, haematologic disorders, endocrinopathies, diabetes mellitus) and morbid obesity. Laboratory methods have been described previously [37].

### Mitochondrial DNA analysis

Mitochondrial haplogroups were assessed as described in our recent studies [38], [39]. Haplogroups that could not be assigned to one of the nine major European haplogroups by their single nucleotide polymorphism (SNP) combination were designated as “others”. The haplogroup frequency in 1527 individuals of the control cohort, in 487 subjects of the CAD group, and in 227 patients with T2DM from Graz were reported previously [29].

CR sequences were analysed between nucleotide positions 16147 and 500, as described previously [38]. In patients with T2DM from Salzburg (n = 279), only the T16189C polymorphism and the poly-C tract were analysed.

### Statistical analysis

Frequencies of all CR polymorphisms and of mitochondrial haplogroups were tested for independence for disease state by using Pearson chi-square statistics and Fisher's exact test as appropriate. Only haplogroups and polymorphisms with a frequency higher than 5% were subjected to further statistical analysis. A p-value <0.05 was considered statistically significant. Association of T16189C and the poly-C tract with T2DM and CAD was corrected for age, sex and body mass index (BMI) by logistic regression analysis. All analyses were performed using SPSS 16.0 (SPSS GmbH, Germany).

## Supporting Information

Table S1 CR polymorphisms with a frequency greater 5% in controls and patients with T2DM recruited at the University Hospital Graz.
(DOC)

Table S2 CR polymorphisms with a frequency greater 5% in controls and patients with CAD.
(DOC)
