# Supplementary material for: The Mitochondrial T16189C Polymorphism Is Associated with Coronary Artery Disease in Middle European Populations
Source: PLoS One. 2011 Jan 26;6(1):e16455. doi: 10.1371/journal.pone.0016455 (PMC3027676; doi:10.1371/journal.pone.0016455)
Supplement: Table S1 — CR polymorphisms with a frequency greater 5% in controls and patients with T2DM recruited at the University Hospital Graz. (DOC) [file pone.0016455.s001.doc]

**Table S1.** CR polymorphisms with a frequency greater 5% in controls and patients with T2DM recruited at the University Hospital Graz.

| Polymorphism  in mtDNA  control region | Frequency (%) in controls  (1481) | n^b^ | Frequency (%) in patients with T2DM^a^ (226) | n^b^ | P-Value^c^ | Odds Ratio (95% CI^d^) |
| --- | --- | --- | --- | --- | --- | --- |
| A 16183 C | 2.30 | 34 | 8.41 | 19 | <0.005^e^ | 3.906 (2.2-7.0) |
| T 16189 C | 11.82 | 175 | 20.80 | 47 | <0.005 | 1.960 (1.4-2.8) |
| C 16192 T | 6.01 | 89 | 4.87 | 11 | 0.496 |  |
| C 16223 T | 5.94 | 88 | 7.52 | 17 | 0.357 |  |
| T 16224 C | 7.77 | 115 | 3.98 | 9 | 0.041^e^ | 0.493 (0.2-1.0) |
| C 16256 T | 6.14 | 91 | 6.19 | 14 | 0.977 |  |
| C 16270 T | 8.04 | 119 | 7.96 | 18 | 0.971 |  |
| C 16294 T | 9.18 | 136 | 12.39 | 28 | 0.128 |  |
| C 16296 T | 6.08 | 90 | 3.54 | 8 | 0.127 |  |
| T 16304 C | 7.77 | 115 | 8.41 | 19 | 0.738 |  |
| T 16311 C | 14.04 | 208 | 13.27 | 30 | 0.756 |  |
| T 16362 C | 6.89 | 102 | 9.29 | 21 | 0.193 |  |
| T 16519 C | 65.63 | 972 | 61.50 | 139 | 0.225 |  |
| A 73 G | 54.42 | 806 | 52.21 | 118 | 0.535 |  |
| T 146 C | 9.32 | 138 | 7.08 | 16 | 0.274 |  |
| C 150 T | 11.55 | 171 | 11.50 | 26 | 0.985 |  |
| T 152 C | 22.82 | 338 | 22.57 | 51 | 0.932 |  |
| G 185 A | 5.74 | 85 | 7.08 | 16 | 0.426 |  |
| T 195 C | 16.95 | 251 | 22.12 | 50 | 0.057 |  |
| G 228 A | 5.94 | 88 | 6.19 | 14 | 0.881 |  |
| A 263 G | 98.78 | 1463 | 99.12 | 224 | 1.000 |  |
| C 295 T | 10.67 | 158 | 11.06 | 25 | 0.859 |  |
| A 302 C-Ins | 37.68 | 558 | 37.61 | 85 | 0.985 |  |
| A 302 CC-Ins | 11.88 | 176 | 8.41 | 19 | 0.126 |  |
| T 310 C-Ins | 96.83 | 1434 | 95.13 | 215 | 0.191 |  |
| C 456 T | 3.24 | 48 | 5.75 | 13 | 0.058 |  |
| C 462 T | 8.44 | 125 | 6.64 | 15 | 0.357 |  |
| T 489 C | 11.68 | 173 | 11.50 | 26 | 0.938 |  |

^a^T2DM = type 2 diabetes mellitus.

^b^n = Number of individuals with the respective polymorphism.

^c^P-Value: Pearson chi-square or Fisher’s exact test, respectively.

^d^CI = Confidence Interval.

^e^Polymorphism in linkage with T16189C.
