# Supplementary material for: The Mitochondrial T16189C Polymorphism Is Associated with Coronary Artery Disease in Middle European Populations
Source: PLoS One. 2011 Jan 26;6(1):e16455. doi: 10.1371/journal.pone.0016455 (PMC3027676; doi:10.1371/journal.pone.0016455)
Supplement: Table S2 — CR polymorphisms with a frequency greater 5% in controls and patients with CAD. (DOC) [file pone.0016455.s002.doc]

**Table S2.** CR polymorphisms with a frequency greater 5% in controls and patients with CAD.

| Polymorphism  in mtDNA  control region | Frequency (%)  in controls  (1481) | n^b^ | Frequency (%)  in patients with CAD^a^ (482) | n^b^ | P-Value^c^ | Odds Ratio (95% CI^d^) |
| --- | --- | --- | --- | --- | --- | --- |
| A 16183 C | 2.30 | 34 | 6.43 | 31 | <0.005^e^ | 2.925 (1.8-4.8) |
| T 16189 C | 11.82 | 175 | 21.58 | 104 | <0.005 005 | 2.053 (1.6-2.7) |
| C 16192 T | 6.01 | 89 | 5.39 | 26 | 0.617 |  |
| C 16223 T | 5.94 | 88 | 7.88 | 38 | 0.131 |  |
| T 16224 C | 7.77 | 115 | 5.39 | 26 | 0.080 |  |
| C 16256 T | 6.14 | 91 | 7.05 | 34 | 0.478 |  |
| C 16261 T | 4.25 | 63 | 5.19 | 25 | 0.390 |  |
| C 16270 T | 8.04 | 119 | 9.96 | 48 | 0.189 |  |
| C 16294 T | 9.18 | 136 | 15.35 | 74 | <0.005^e^ | 1.794 (1.3-2.4) |
| C 16296 T | 6.08 | 90 | 7.88 | 38 | 0.163 |  |
| T 16304 C | 7.77 | 115 | 11.20 | 54 | 0.019^f^ | 1.499 (1.1-2.1) |
| T 16311 C | 14.04 | 208 | 13.90 | 67 | 0.937 |  |
| T 16356 C | 4.39 | 65 | 5.19 | 25 | 0.467 |  |
| T 16362 C | 6.89 | 102 | 9.13 | 44 | 0.103 |  |
| A 16399 G | 4.12 | 61 | 5.39 | 26 | 0.237 |  |
| T 16519 C | 65.63 | 972 | 64.94 | 313 | 0.781 |  |
| A 73 G | 54.42 | 806 | 59.54 | 287 | 0.049^e^ | 1.233 (1.0-1.5) |
| T 146 C | 9.32 | 138 | 7.47 | 36 | 0.215 |  |
| C 150 T | 11.55 | 171 | 11.41 | 55 | 0.935 |  |
| T 152 C | 22.82 | 338 | 23.03 | 111 | 0.925 |  |
| G 185 A | 5.74 | 85 | 7.68 | 37 | 0.126 |  |
| T 195 C | 16.95 | 251 | 21.99 | 106 | 0.013^e^ | 1.381 (1.1-1.8) |
| G 228 A | 5.94 | 88 | 6.65 | 32 | 0.572 |  |
| A 263 G | 98.78 | 1463 | 99.58 | 479 | 0.190 |  |
| C 295 T | 10.52 | 161 | 11.02 | 53 | 0.829 |  |
| A 302 C-Ins | 37.68 | 558 | 38.88 | 187 | 0.638 |  |
| A 302 CC-Ins | 11.88 | 176 | 13.10 | 63 | 0.479 |  |
| T 310 C-Ins | 96.83 | 1434 | 96.26 | 463 | 0.545 |  |
| C 462 T | 8.44 | 125 | 8.32 | 40 | 0.932 |  |
| T 489 C | 11.68 | 173 | 11.23 | 54 | 0.786 |  |

^a^CAD = coronary artery disease.

^b^n = Number of individuals with the respective polymorphism.

^c^P-Value: Pearson chi-square or Fisher’s exact test, respectively.

^d^CI = Confidence Interval.

^e^Polymorphism in linkage with T16189C.

^f^Polymorphism in linkage with haplogroup T.
